# Hypertension, a Neglected Disease in Rural and Urban Areas in Moramanga, Madagascar

**DOI:** 10.1371/journal.pone.0137408

**Published:** 2015-09-10

**Authors:** Rila Ratovoson, Ony Rabarisoa Rasetarinera, Ionimalala Andrianantenaina, Christophe Rogier, Patrice Piola, Pierre Pacaud

**Affiliations:** 1 Pasteur Institute of Madagascar, PO Box: 1274 Ambatofotsikely, Antananarivo, Madagascar; 2 Faculty of Medicine, Antananarivo University, Antananarivo, Madagascar; 3 Unité de Recherche sur les Maladies Infectieuses et Tropicales Émergentes (URMITE), UMR 6236, 27 boulevard Jean Moulin, 13385, Marseille Cedex 05, France; 4 Institute for Biomedical Research of the French Armed Forces (IRBA), BP 73, 91223, Brétigny-Sur-Orge Cedex, France; 5 Inserm, UMR_S1087, University of Nantes, CHU Nantes, l'Institut du thorax, Nantes, F-44000, France; The University of Manchester, UNITED KINGDOM

## Abstract

**Background:**

Hypertension is one of the main risk factors of cardiovascular diseases. In Madagascar, studies on hypertension in urban and rural communities are scarce.

**Objectives:**

The aim of this study was to determine the prevalence of hypertension and identify associated risk factors in adults living in a health and demographic system in Moramanga, Madagascar.

**Methods:**

The study included people aged 15 years old and above living in a health and demographic system in Moramanga. A household census was performed in 2012 to enumerate the population in 3 communities in Moramanga. In addition to the questionnaire used in the initial census, a standardized questionnaire and blood pressure were taken twice after 5 and 10 minutes of rest. In urban areas, heights and weights were also measured to calculate the body mass index.

**Results:**

There were 3621 and 4010 participants respectively in rural and urban areas. Prevalence of hypertension in rural population was 27.0% (IC95% [25.6–28.5]) and 29.7% (IC95% [28.3–31.1]) in urban population. Among hypertensive subjects, 1.7% (17/979) and 5.3% (64/1191) were on antihypertensive treatment for at least 1 month before the survey in rural and urban population, respectively. In rural areas, increasing age (65 years and older vs 18–25 years OR = 11.81, IC95% [7.79–18.07]), giving more than 3 positive responses to the usual risks factors of hypertension (OR = 1.67, IC95% [1.14–2.42]) and singles in comparison with married people (OR = 1.61, IC95% [1.20–2.17]) were associated to hypertension in a logistic regression model. In urban areas, increasing age (65 years and older vs 18–25 years OR = 37.54, IC95% [24.81–57.92]), more than 3 positive responses to the usual risks of hypertension (OR = 3.47, IC95% [2.58–4.67]) and obesity (OR = 2.45, IC95% [1.56–3.87]) were found as risk factors.

**Conclusion:**

Hypertension is highly prevalent in rural areas although it is significantly less treated. As a result, a major epidemic of cardiovascular diseases is at risk in Madagascar’s progressively aging society.

## Introduction

Cardiovascular diseases remain a major worldwide public health problem, affecting both developed and developing countries [1]. Approximately 80% of deaths in low-middle income countries were due to the complication of hypertension [2]. In the past decades, hypertension has become the fifth most important risk factor for deficient health in developing countries [3]. Because of the change in lifestyle and urbanization, many African countries now have an increasing prevalence of hypertension [3–15]. Madagascar is not spared from this problem. Recent study showed that Antananarivo now faces a double burden of communicable and non communicable diseases, including cardio-vascular diseases [16]. Some studies on hypertension have been conducted in health facilities in Madagascar to estimate its prevalence, but until now none has been carried out in the community in both urban and rural areas [17, 18]. Our study aimed to estimate the prevalence and identify the risk factors of hypertension among adults aged 15 years and older in an urban and rural area at Moramanga, a district located at 100 km from the capital.

## Materials and Methods

### Study population and setting

A cross-sectional study was conducted among adults aged 15 years and above in the urban and 2 rural communities of Moramanga district. These three communities are part of a Health and Demographic Surveillance Site of Moramanga (HDSS Moramanga) implemented by Institut Pasteur de Madagascar in 2010. Moramanga area is a crossroads between the capital and the largest port in eastern Madagascar. The census established that the local population was 37,031 inhabitants in urban area and 32,717 inhabitants in the 2 rural communities. According to the Demographic and Health Survey 2008–2009 of Madagascar, 52.9% of the population was aged 15 years and above [19]. Half of the population in Moramanga is engaged in agricultural activities and cattle farming, 20% have no determined activity, 12% are public officials and the remaining is divided among employees of private companies and artisans.

### Community census

Institut Pasteur de Madagascar has carried out a longitudinal demographic surveillance on the population of three communities in the district of Moramanga (Moramanga urban community, Ampasimpotsy and Ambohibary rural communities). A community-wide household level census was performed in the HDSS Moramanga. Trained staff with the support of local leaders conducted a door to-door census. When a visit was made, the head of the household or his wife provided informed consent to collect demographic information about all household members. A unique household identifier was given to each household to identify inhabitants in the HDSS.

### Data collection

In addition to the questionnaire used for the census, participants in the hypertension survey were also submitted to another standardized questionnaire on risk factors for hypertension. The questionnaire administration and blood pressure measurements were carried out by trained interviewers from February to May 2013 for rural populations and from August 2013 to January 2014 for urban populations. In urban areas, physical measurements were performed and included the height, weight, and blood pressure. Data collection was conducted every day including weekends and evenings when people were more likely to be found at home.

#### Blood pressure measurement and anthropometry

Blood pressure (BP) was measured using automated digital blood pressure monitor, OMRON M3, with appropriate cuff sizes. Two BP measurements were taken after 5 minutes rest and at least 5 minutes apart. The average of two readings of systolic BP and diastolic BP were used to describe the BP for each participant. Hypertension was defined as mean systolic BP ≥ 140 mmHg or mean diastolic BP ≥ 90 mmHg [20]. Self reported use of antihypertensive medication prescribed by physician in the four weeks preceding the study was also defined as hypertensive subject. The reporting of these patients treated for at least one month was verified from health booklets or the presence of drugs at the time of investigation.

Anthropometric measurements were not available in rural areas. Height and weight were recorded in urban areas only. Height was measured to the nearest 0.1 cm after removal shoes. Weight was measured to the nearest kilogram after removal shoes and heavy clothing. Body mass index (BMI) was calculated to determine proportion of obese (≥30 kg/m²) in urban areas.

#### Sampling

Hypertension study took place simultaneously with the census in rural areas. Four fokontany (the lowest administrative subdivision in Madagascar) out of 5 were included in Ampasimpotsy and 3 fokontany out of 12 in Ambohibary community. In urban areas, the study took place at the end of the census of each village to allow the random sampling of participants. From each fokontany, in urban areas, the sample size was calculated under the assumption that the prevalence was at 22%, with a confidence level at 95% and a precision at 0.04. Village populations range from 957 to 4529 inhabitants. As there were 13 urban, the number of participants ranged from 289 to 378 per fokontany. All 13 fokontany were included in urban areas.

### Data analysis

Data was collected by trained interviewers through a tablet PC data entry template. As the methodology used in urban and rural area was not similar, we conducted analysis separately. Analyses were performed with R software on three levels: descriptive, univariate and multivariate [21].

Regarding risk factors, we added the number of positive responses to known risk factors for hypertension (history of blood pressure above 130 mmHg for systolic BP (SBP) or 80 mmHg for diastolic BP (DBP), family history of hypertension, recent weight gain, inactivity, salt diet, smoking, alcoholic habit). The risk assessment has been classified into 3 groups: high risk if more than three positive responses, moderate if 2–3 positive responses, and low risk if no or only one positive response. The habitat characteristics and household assets collected in the census were used to assess socioeconomic status using principal components analysis [22, 23]. In rural areas, number of bedroom, possession of farm animals (chicken, ducks, and cattle), and possession of land to cultivate (gardens, orchards) and cart were included. In urban area, habitat characteristics (number of bedroom, type of floor, lighting, fuel used for cooking, type of toilet, drinking water supply), household asset (possession of radio, television, mobile phone, fridge, sewing machine, motor vehicle), and possession of animals (chicken and dog) were included (S1 File). The coefficients were calculated by using the loading of the first principal component and scores was obtained on the coded value of the variables mentioned. Households were classified into 5 classes by quintile: poorest, poor, middle, rich, richest [23].

Means and standard deviations (SD) were calculated for quantitative variables and proportions for categorical variables. The Chi-square test and Fisher’s exact test were used for univariate analysis. P-values < 0.05 were considered to be statistically significant.

Explanatory variables associated with a p-value less than 0.15 were analyzed by logistic regression to investigate the confounding factors. A multivariate logistic regression model was employed with stepwise backward elimination of non-significant variables, with hypertension status as the dependent variable. P values < 0.05 were considered to be statistically significant.

### Ethics statement

Written informed consent was obtained from participant before enrolment on the hypertension study. The study was approved by the Ethics Committee of the Ministry of Public Health of Madagascar (Number 020-MSANP/CE– 08/02/2013). For minors, written informed consent was obtained from parents or guardians on behalf of the minors enrolled in the study.

## Results

### Study sample characteristics

In rural area, 3708 persons were found at home and consented to participate in the study. Eighty seven (2.3%) persons were excluded from analysis because of missing data. In urban areas, 4025 persons consented to participate in the study and 4010 (99.6%) were analyzed (S2 File). Both in urban and rural areas, more females were included because they were more likely to be found at home compared to men. Of the 3621 analyzed in rural area, 53.6% were females and 56.2% of 4010 in urban areas. In rural area, mean age and SD of participants was 35.8±15.3 years old, age maximum was 100.3 years old and the interquartile range was 23.2–45.6 years old. Among the rural participants, 75.4% had an activity with ongoing income, 2.3% were retired or enable to work, and 4.5% were students, 13.2% were housewives or househusbands and 4.5% were jobless. In urban area, mean age of participants was 36.1±15.1 years old, age maximum was 100.6 years old and the interquartile range was 23.5–46.0 years old. Among the participants, 52.7% had an activity, 6.1% were retired or enable to work, 13.6% were students, 22.3% were housewives/househusbands and 5.3% were jobless. Table 1 illustrates demographic and clinical characteristics of studied population in rural and urban areas.

**Table 1:** Demographic characteristics of study participants in urban and rural area.

	Rural(N = 3621)		Urban(N = 4010)	
**Sex**				
Male	1679	(46.4%)	1756	(43.8%)
Female	1942	(53.6%)	2254	(56.2%)
**Age (years)**				
15–17	317	(8.8%)	321	(8.0%)
18–25	779	(21.5%)	842	(21.0%)
26–35	886	(24.5%)	1032	(25.7%)
36–45	701	(19.4%)	766	(19.1%)
46–55	464	(12.8%)	491	(12.2%)
56–65	300	(8.3%)	369	(9.2%)
More than 65 years old	174	(4.8%)	189	(4.7%)
**Occupation**				
Activity with ongoing income	2728	(75.3%)	2114	(52.7%)
Jobless	162	(4.5%)	213	(5.3%)
Housewives/househusbands	479	(13.3%)	895	(22.3%)
Students	166	(4.6%)	547	(13.6%)
Retired or enable (for other reason…)	83	(2.3%)	241	(6.1%)
Unknown	3	(0.0%)	-	
**Education**				
Never in school	311	(8.6%)	98	(2.4%)
Previously but not currently in school	3148	(86.9%)	3368	(84.0%)
Currently in school	162	(4.5%)	544	(13.6%)
**Marital status**				
Single	706	(19.5%)	941	(23.5%)
Married	1747	(48.2%)	1970	(49.1%)
Cohabitation	730	(20.2%)	612	(15.3%)
Divorced	211	(5.8%)	206	(5.1%)
Separated	56	(1.5%)	75	(1.9%)
Widower	170	(4.7%)	205	(5.1%)
Unknown	1	(0.0%)	1	(0.0%)
**Socio-economic level** *				
Poorest	616	(17.0%)	749	(19.0%)
Poor	663	(18.3%)	748	(18.9%)
Middle	710	(19.6%)	755	(19.1%)
Rich	772	(21.3%)	841	(21.3%)
Richest	860	(23.8%)	858	(21.7%)

*59 missing values in urban area.

### Prevalence of hypertension, treatment of hypertension

As the methods used in urban and rural area was not similar, the prevalence of hypertension in each area was calculated separately. It was 27.0% (979/3621) CI _95%_ [25.6%-28.5%] in rural area and 29.7% (1191/4010) in urban area CI _95%_ [28.3%-31.1%]. Patients on antihypertensive treatment for at least 1 month were 5.4% (64/1191) CI _95%_ [4.1%-6.8%] in urban and 1.7% (17/979) CI _95%_ [1.0%-2.8%] in rural areas. According to blood pressure level, 31.3% (20/64) and 47.1% (8/17) of patients receiving antihypertensive treatment for at least 1 month respectively in urban and rural areas had very high blood pressure (Table 2).

**Table 2:** Distribution of patients on antihypertensive treatment for at least 1 month in urban and rural areas according to the level of blood pressure.

Level of BP	Rural			Urban		
	On treatment n (%)		Total	On treatment n(%)		Total
Non hypertensive (SBP<140mmHg and DBP<90mmHg)	3	(17.6%)	2645	14	(21.9%)	2833
SBP [140–150]mmHg or DBP [90–100]mmHg	4	(23.5%)	471	10	(15.7%)	538
SBP [150–180]mmHg or DBP [100–110]mmHg	2	(11.8%)	375	20	(31.2%)	408
SBP>180 mmHg or DBP>110mmHg	8	(47.1%)	130	20	(31.2%)	231
Total	17	(100%)	3621	64	(100%)	4010

### Sociodemographic and risk factors associated with hypertension

#### Known Risk factors

In the present study, the exposure to major known risk factors was asked. In order to estimate the level of risk, we summed the number of positive responses to the questions about the exposure to known risk factors for hypertension (history of blood pressure above 130 mmHg for systolic BP (SBP) or 80 mmHg for diastolic BP (DBP), family history of hypertension, recent weight gain, inactivity, salt diet, smoking, alcoholic habit). The risk level has been categorized into 3 ordinal groups: high risk if more than three positive responses, moderate if 2–3 positive responses, and low risk if no or only one positive response (Table 3).

**Table 3:** Distribution on known risk factors and classification of risk in urban and rural areas.

	Rural			Urban		
	Hypertensive n (%)		Total	Hypertensive n (%)		Total
History of SBP>130mmHg or DBP>80mmHg	253	(50.3%)	503	561	(61.4%)	913
Family history of hypertension	381	(26.8%)	1422	698	(33.3%)	2093
Recent weight gain	184	(24.1%)	765	352	(30.6%)	1152
Physical inactivity	76	(29.5%)	258	164	(31.7%)	518
Salt diet	262	(25.5%)	1029	290	(26.7%)	1087
Smoking habit	130	(30.6%)	425	206	(32.8%)	628
Alcoholic habit	229	(29.9%)	766	190	(38.9%)	489
**Risks**						
*** Low***	***494***	***(24*.*3%)***	***2035***	***387***	***(21*.*2%)***	***1822***
*** Moderate***	***433***	***(30*.*2%)***	***1435***	***669***	***(34*.*9%)***	***1915***
*** High***	***52***	***(34*.*4%)***	***151***	***135***	***(49*.*6%)***	***272***

In rural areas, there was no significant association of hypertension with sex or socioeconomic level. The prevalence rate increased with age. In univariate analysis, people aged 36–45 years old had twice the risk to be hypertensive than those aged 18–25 years old (crude odds ratio cOR = 2.11 CI_95%_ [1.64–2.74]) and the risk of those aged more than 65 years old was 10 times higher (cOR = 10.6 CI_95%_ [7.39–15.47]) (Table 4). Marital status, activity and education were also significantly associated with hypertension (p<0.001).

**Table 4:** Risk factors associated with hypertension in rural area.

	Hypertensive		Total	p	cOR	CI _95%_	aOR	CI _95%_
	n	(%)	N					
**Age (years)**				<0.0001				
15–17	27	(8.5%)	317		0.52	0.33–0.79	0.45	0.28–0.71
18–25	118	(15.1%)	779		1	-	1	-
26–35	184	(20.8%)	886		1.47	1.14–1.89	1.61	1.23–2.14
36–45	192	(27.4%)	701		2.11	1.63–2.73	2.36	1.77–3.16
46–55	179	(38.6%)	464		3.52	2.68–4.62	4.05	2.99–5.51
56–65	165	(55.0%)	300		6.85	5.08–9.26	7.76	5.56–10.90
More than 65 years old	114	(65.5%)	174		10.64	7.39–15.47	11.81	7.79–18.07
**Sex**				0.67				
Male	460	(27.4%)	1679					
Female	519	(26.7%)	1942					
**Occupation** *				<0.0001				
Activity with ongoing income	761	(27.9%)	2731		1	1		
Jobless	32	(19.8%)	162		0.637	0.42–0.93		
Housewives/househusbands	121	(25.3%)	479		0.875	0.69–1.09		
Students	16	(9.9%)	162		0.284	0.16–0.46		
Retired or enable (for other reason…)	47	(56.0%)	84		3.288	2.12–5.12		
**Education**				<0.0001				
Never in school	109	(35.0%)	311		1	1		
Previously but not currently in school	854	(27.1%)	3148		0.69	0.541–0.884		
Currently in school	16	(9.9%)	162		0.20	0.112–0.349		
**Marital status** **				<0.0001				
Single	116	(16.4%)	706		1	1	1	-
Married	469	(26.8%)	1747		1.87	1.49–2.35	0.62	0.46–0.83
Cohabitation	200	(27.4%)	730		1.91	1.49–2.45	0.77	0.56–1.06
Divorced	81	(38.4%)	211		3.17	2.25–4.46	1.01	0.67–1.51
Separated	15	(26.8%)	56		1.86	0.97–3.40	0.62	0.30–1.20
Widower	97	(57.1%)	170		6.76	4.71–9.75	0.89	0.56–1.41
**Socio-economic level**				0.6				
Poorest	162	(26.3%)	616					
Poor	183	(27.6%)	663					
Middle	177	(24.9%)	710					
Rich	214	(27.7%)	772					
Richest	243	(28.3%)	860					
**Risks**				0.0001				
Low	494	(24.3%)	2035		1	1	1	-
Moderate	433	(30.2%)	1435		1.35	1.15–1.57	1.38	1.17–1.62
High	52	(34.4%)	151		1.64	1.14–2.31	1.67	1.14–2.42
**Dizziness**				0.66				
Yes	497	(27.4%)	1815					
No	482	(26.7%)	1806					
**Tinnitus**				0.4				
Yes	515	(27.7%)	1862					
No	464	(26.4%)	1759					
**Headache on waking**				0.006				
Yes	362	(29.9%)	1209		1	1	1	-
No	617	(25.6%)	2412		0.80	0.69–0.93	0.81	0.69–0.96
**Epistaxis**				0.4				
Yes	110	(25.3%)	435					
No	869	(27.3%)	3186					
**Diabetes**				0.16^#^				
Yes	8	(44.4%)	18					
No	227	(25.9%)	878					
Unknown	744	(27.3%)	2725					

*3 missing values,

**1 missing value,

^#^fisher exact test.

Assessing risks through positive responses to questions about the exposure to known risk factors of hypertension, the prevalence increased with the number of positive responses: compared to low risk (no or 1 positive response), crude odds ratio (cOR) was equal to 1.64 CI _95%_ [1.15–2.31] for people who had more than 3 positive responses.

In multivariate analysis, increasing age, marital status, exposure to the known risks factors and the presence of headache on waking were independently and significantly associated with hypertension (Table 4). Adjusted to other variables, the elderly were more likely to have high BP compared to young adults between 18–25 years with adjusted odds ratio (aOR = 11.81 CI _95%_ [7.79–18.07]). Single people were more format risk of high BP than married people independently of the other factors (aOR = 1.61, IC_95%_ [1.20–2.17]).

In urban area, the prevalence rate increased with age. A statistically significant association was found between hypertension and the following variables in univariate analysis: employment, education, marital status, socioeconomic status, positive responses to known risks, diabetes and BMI. No significant association between high BP and other variables such as sex and clinical signs as dizziness, tinnitus, headache, and epistaxis was observed (Table 5). In urban area, people aged 36–45 years old had five-fold risk to be hypertensive than those aged 18–25 years old (cOR = 5.58 CI _95%_ [4.25–7.39]). People who declared not to be diabetic had lower risk of hypertension (cOR = 0.35 CI _95%_ [0.19–0.62]). In multivariate analysis, increasing age, positive responses to the questions about the exposure to known risk factors and obesity were associated with hypertension (Table 5). Adjusted for age and risk factors of hypertension, obese participants were more likely to be hypertensive than others (aOR = 2.45 CI _95%_ (1.56–3.86]).

**Table 5:** Risk factors associated with hypertension in urban area.

	Hypertensive		Total	p	cOR	CI _95%_	aOR	CI _95%_
	n	(%)	N					
**Age (years)**				<0.0001				
15–17	20	(6.2%)	301		0.65	0.38–1.06	0.71	0.41–1.16
18–25	78	(9.3%)	764		1	1	1	-
26–35	198	(19.2%)	1032		2.32	1.76–3.09	2.24	1.69–2.99
36–45	278	(36.3%)	766		5.58	4.25–7.39	5.19	3.94–6.91
46–55	239	(48.7%)	491		9.29	6.96–12.51	8.75	6.52–11.86
56–65	232	(62.9%)	369		16.59	12.17–22.83	16.37	11.91–22.73
More than 65 years old	146	(77.2%)	189		33.26	22.22–50.73	37.54	24.81–57.92
**Sex**				0.14				
Male	543	(30.9%)	1756					
Female	648	(28.7%)	2254					
**Occupation**				<0.0001				
Activity with ongoing income	672	(31.8%)	2114		1	1		
Jobless	41	(19.2%)	213		0.51	0.35–0.72		
Housewives/househusbands	276	(30.8%)	895		0.96	0.80–1.13		
Students	46	(8.4%)	547		0.19	0.14–0.26		
Retired or enable (for other reason…)	156	(64.7%)	241		3.94	2.98–5.22		
**Education**				<0.0001				
Never in school	38	(38.8%)	98		1	1		
Previously but not currently in school	1109	(32.9%)	3368		0.775	0.51–1.18		
Currently in school	44	(8.1%)	544		0.139	0.08–0.23		
**Marital status** *				<0.0001				
Single	124	(13.2%)	941		1	1		
Married	678	(34.4%)	1970		3.46	2.81–4.28		
Cohabitation	162	(26.5%)	612		2.37	1.82–3.08		
Divorced	88	(42.7%)	206		4.91	3.51–6.86		
Separated	20	(26.7%)	75		2.39	1.36–4.07		
Widower	119	(58.0%)	205		9.12	6.53–12.79		
**Socio-economic level** **				0.001				
Poorest	218	(29.1%)	749		1	1		
Poor	195	(26.1%)	748		0.858	0.68–1.07		
Middle	200	(26.5%)	755		0.877	0.70–1.09		
Rich	264	(31.4%)	841		1.114	0.89–1.38		
Richest	294	(34.3%)	858		1.269	1.02–1.56		
**Risks**				<0.0001				
Low	387	(21.2%)	1822		1	1	1	1
Moderate	669	(34.9%)	1915		1.99	1.72–2.30	2.07	1.75–2.44
High	135	(49.6%)	272		3.65	2.80–4.75	3.47	2.58–4.67
**Dizziness**				0.231				
Yes	443	(28.6%)	1550					
No	748	(30.4%)	2460					
**Tinnitus**				0.159				
Yes	460	(31.1%)	1481					
No	731	(28.9%)	2529					
**Headache on waking**				0.9				
Yes	351	(29.5%)	1189					
No	840	(29.8%)	2821					
**Epistaxis**				0.9				
Yes	136	(29.4%)	463					
No	1055	(29.7%)	3547					
**Diabetes**				0.0001				
Yes	28	(56.0%)	50		1	1		
No	359	(30.8%)	1167		0.35	0.19–0.62		
Unknown	804	(28.8%)	2793		0.32	0.17–0.55		
**BMI** ***				<0.0001				
Yes	56	(57.1%)	98		3.26	2.17–4.91	2.45	1.56–3.87
No	1135	(29.0%)	3910		1	1	1	1

*1 missing value,

**59 missing values,

***2 missing values.

## Discussion

To our knowledge, this is the first study on hypertension in the community in Madagascar.

In a population based survey of adults aged 15 years and over conducted in the district of Moramanga in Madagascar, more than a quarter of the population had hypertension. We decided to include people aged 15 and older considering that it is from this age that patients are no more considered as children, *i*.*e*. not admitted to the pediatric ward but in internal adults medical wards at the hospital in Moramanga.

The prevalence that we found in rural (27.0%) and urban (29.7%) areas were consistent with studies conducted in Sub-Saharan Africa which show prevalence of hypertension ranging from 20% to 50% [1, 7, 8, 11, 14, 24]. However, this comparison should be made carefully because age groups used are different according to studies. Most epidemiological studies show the prevalence of hypertension higher in urban than in rural areas in the same country. Our result adds those studies showing that the prevalence of hypertension in sub-Saharan Africa is similar to those in developed countries [1, 4]. There is compelling need to change the dogmatic notion that in developing countries main health problems are solely communicable diseases and non-communicable diseases are merely problems of developed countries [3, 25].

Furthermore, among people defined as hypertensive, we found very low rates of therapy (5.4% in urban and 1.7% in rural). Treatment of hypertension and optimal control of BP are minority in developed and developing countries [1, 26, 27]. Majority of people with hypertension are not aware of his BP. Even among those on therapy, very few had BP below 140/90 mmHg (17.6% in rural and 21.9% in urban). A survey from Tanzania showed that only 1% of hypertensive patients had BP readings less than 140/90mmHg [28]. This lack of hypertension awareness may be due to a flaw in the health system as in most of sub-Saharan countries [6]. In the study area, only 5 public health centers deserve the two rural communities composed of 32 717 inhabitants. Level of awareness, treatment and control of hypertension are alarming low in developing countries [3]. One reason of this may be the availability of basic instruments such as a simple and functional sphygmomanometer mainly in remote place like rural area. Furthermore, health staff are accustomed to focus on the more dramatic complaints of the numerous patients with infectious diseases, obstetric illness or trauma before thinking about hypertension [3]. According to World Health Organization, the mortality rate for non-communicable diseases is nearly twice higher than that of communicable diseases in Madagascar (706 vs 408/100 000 hab.) in 2008; cardiovascular diseases and diabetes accounts for deaths of 289 /100 000 adults aged between 30–70 years old [29]. These data suggest that the management of hypertension remains difficult especially in remote places of rural areas. This poor level of awareness associated with high prevalence of hypertension require the development of appropriate and cost effective strategies for prevention and treatment of hypertension [25].

In addition, the present study revealed that prevalence of hypertension among people aged from 36 years old is high (27.4% in rural and 36.3% in urban). This result confirms other studies showing that hypertension is increasingly affecting young people in developing countries [6, 30]. As showed by other studies, the risk of hypertension increased with age [1, 6–8, 11, 14]. A prospective study in Tanzania found that hypertension-related diseases accounted for 15% of all deaths and half of them occurred in patients younger than 65 years [31]. So it is clear that the lack of appropriate care of hypertension in poor countries contributes significantly to the change in life expectancy and productivity.

In the present study, the prevalence of hypertension was higher among urban resident and among those who were obese. Urbanized areas are favorable to the development of obesity [32]. Although Moramanga is 100 km away from the capital, the results of the present study showed that obesity is also present in this area. Obesity is attributed to changes in dietary and physical activity patterns which are the result of urbanization and societal changes [33]. The by-products of the growing economies dumped at the market in lower prices and affordable motorized transport contribute to gradual increase of obesity in urban area [3]. Furthermore, the surge in obesity is fueled by the overwhelming availability of affordable highly processed and “fast” foods, together with highly refined fats, oils, and carbohydrates served in most eateries and fast food found in the urban areas [34]. In addition, open areas, like parks and public properties are gradually replaced by new buildings and car parks. Young people who used to walk and cycle long distances to work in agricultural fields or semirural towns become accustomed on motorized transport further leading to a higher risk of obesity [3].

In rural areas, the present study did not show a significant association between sex and hypertension. However, married subjects were more protected against high BP compared to others marital status. One explication of this protective effect of marital status would be that in addition to himself, there would also be his wife or husband who takes care of the person. Other studies have shown the influence of marital status in relation to cardiovascular risk factors [35, 36].

Both in rural and urban area, people who were more exposed to known risk factors (personal history of high blood pressure, family history of hypertension, recent weight gain, physical inactivity, salt diet, alcohol-smoking habits) were more likely to be hypertensive. Answers obtained from those questions might be influenced by the results of the measurement of BP, but to avoid this, the interviewers gave the results of respondent’s BP at the end of the interview. Our results suggest that high blood pressure did not spare the rural area in Madagascar. There is an urgent need to improve the knowledge of people about modifiable factors of hypertension that is eviction of a sedentary lifestyle and habit of healthy eating. In certain some developing regions like in Africa, because of no available refrigeration capacity, high concentrations of salt are used for the preparation and the preservation of food. This habit might have contributed to the development of hypertension [3, 32].

Advantages of the present study were the large sample size both in rural and urban area. However, this study has some limitations. The body weight was taken both in urban and rural area but height was taken only in urban areas for logistical reasons.

The sample was not strictly representative, more women and more elderly were found at home, distribution by villages also differ (S1 Table). It is important to consider this limitation in order to generalize the findings of the present study to other population. For pragmatic reason, the two measurements of blood pressure were performed on one occasion which may overestimate the prevalence of hypertension [27]. However, according studies realized with the same process, this should have minimal effects on the results concerning the “within the sample” comparison [6, 27, 37].

In order not to overload the population survey, some risk factors (waist hip ratio, diet survey) were not investigated at this first study whose main objective was to estimate the prevalence of hypertension in Moramanga community. Further studies are needed to investigate risk factors for cardiovascular diseases.

## Conclusion

The risk factors that have been found in the present study do not differ from other studies on hypertension. Hypertension has high prevalence in both rural and urban residents of the region of Moramanga. As a result, a major epidemic of cardio-vascular diseases could be anticipated in the Madagascar’s progressively aging society. This is a major public health problem. Hypertension prevention guidelines, measures for increasing awareness and changes in lifestyles are needed. These measures would result in a lower prevalence of hypertension. Finally, further studies to reveal the hidden burden of hypertension nationwide are necessary.

## Supporting Information

S1 File Data collected in rural and urban areas in Moramanga during the census 2013–2014. (XLSX)

S2 File Data collected in rural and urban areas in Moramanga during the hypertension study, 2013–2014. (XLSX)

S1 Table Structure of rural population compared with the population census by age, sex, and villages in Moramanga. (DOCX)
