# Supplementary material for: Hypertension, a Neglected Disease in Rural and Urban Areas in Moramanga, Madagascar
Source: PLoS One. 2015 Sep 10;10(9):e0137408. doi: 10.1371/journal.pone.0137408 (PMC4565657; doi:10.1371/journal.pone.0137408)
Supplement: S1 Table — (DOCX) [file pone.0137408.s003.docx]

# Supporting information

S1 Table. Structure of rural population compared with the population census by age, sex, and villages in Moramanga.

| Characteristics | Population study | Population in census | Expected size (%) | p |
| --- | --- | --- | --- | --- |
| Sex |  |  |  | <0.0001 |
| Male | 1679 | 2928 | 49.8 |  |
| Female | 1942 | 2949 | 50.2 |  |
| Age |  |  |  | <0.0001 |
| 15-25 | 1096 | 2140 | 36.4 |  |
| 26-35 | 886 | 1351 | 23.0 |  |
| 36-45 | 701 | 1060 | 18.0 |  |
| 46-55 | 464 | 674 | 11.5 |  |
| More than 55 years | 474 | 652 | 11.1 |  |
| Community/Fokontany |  |  |  |  |
| Ambohibary community |  |  |  | <0.0001 |
| Analalava | 566 | 1568 | 26.7 |  |
| Ambohimanatrika | 86 | 349 | 5.9 |  |
| Ankarahara | 937 | 1282 | 21.8 |  |
| Ampasimpotsy community |  |  |  |  |
| Ampasimpotsy gare | 615 | 885 | 15.1 |  |
| Ambatoharanana | 376 | 288 | 4.9 |  |
| Ambodiriana | 568 | 734 | 12.5 |  |
| Amparafara | 473 | 771 | 13.1 |  |
